# Health disparity is a global issue: Understanding Latin America

**DOI:** 10.1002/pul2.12049

**Published:** 2022-02-14

**Authors:** Mardi Gomberg‐Maitland, Vinicio de Jesus Perez

**Affiliations:** ^1^ Department of Medicine, Section of Cardiology George Washington School of Medicine and Health Sciences Washington District of Columbia USA; ^2^ Department of Medicine, Section of Pulmonary and Critical Care Stanford Translational Investigator Program (TIP), Stanford University Medical Center Palo Alto California USA

Inequity in health care is a multifaceted complex global issue not unique to rare disease. Grouping countries into one unified entity is imperfect without a combined political‐strategic goal to improve health. Common disease treatment lacks this equity and patients with rare disease, inherent to the limited prevalence and cost of therapy suffer perhaps more so.

Differences in wealth, access, medical expertise, and political views on the importance and governance of health care are each a component of the disparity. In 1986, members of the United Nations (UN) proclaimed the “Declaration on the Right to Development” and 30 years later these goals expanded with the establishment of the UN Right to Health and the UN Sustainable Development. The goals of “ensuring healthy lives promoting the well‐being for all at all ages” and of “leaving no one behind” deems that all people are entitled to equal quality of health.^1^, ^2^ This is the goal for all physicians, to provide the best care possible and give their patients all the options available to improve their quality of life and ultimately survival. No country is immune to these facets, as seen and magnified unfortunately by the COVID‐19 pandemic. COVID‐19 may in fact delay its goal completion in 2030.^1^, ^2^

As physician–scientists for rare diseases, pulmonary hypertension (PH), and chronic thromboembolic PH, here in the United States, many of the issues faced globally for rare disease exist. There is inequity of health care, access to care, access to medication, and wealth distribution in US geographic regions, states, cities, down to the community level. Although the US has one health system, insurance companies and states can limit access to advance medications and devices. This editorial is not aimed to tackle all these issues and provide answers but to highlight the issues faced by Latin American Country providers and patients with orphan disease, such as pulmonary arterial hypertension and chronic thromboembolic PH.

The authors report the findings of their Latin America collaborative workshop (April 2019) to identify usual care in each country for PH/chronic thromboembolic pulmonary hypertension (CTEPH) and understand the barriers faced by each country.^3^ Information gathering is an important step to identify gaps in health. The authors describe clinic availability, physician expertise, diagnostic testing, and therapeutic options, such as drug dosing, in Argentina, Brazil, Colombia, and Mexico. Understanding that this is only a small fraction of Latin American countries, although encompassing most of the population, their data collection and potential registry do provide insight into the existing fragmented care. All countries do not have all currently approved options for PH/CTEPH and also present differences in PH epidemiology.^4^ The current guidelines and consensus statements do not guarantee and often have little bearing on clinical practice patterns and the availability of therapies. Economics and geographic desirability limit care in the rural areas of all countries.

A major observation raised in the workshop is the challenges of applying the 2018 World Symposium for Pulmonary Hypertension (Nice, France) proceedings to PH treatment in Latin America. Limited medication access and geographic barriers often prevent physicians from aggressively treating patients at high risk of mortality. This problem is compounded by the high cost of the drugs, limited access to high‐quality generic formulations, and lack of coverage by national health programs. Prioritizing education and patient advocacy are recognized as important strategies to maximize the impact of available therapies through early initiation and through discussion with government agencies to include more PH drugs in the formulary. Besides education and therapeutics, the workshop members highlight the importance of conducting clinical, basic, and epidemiological research on PH in Latin America since it is likely that there are unique determinants that could influence treatment response and survival.

The paper is limited by its narrow focus and lack of clear direction for the patients and physicians on the “how” to improve care. The authors did not broach the topic of clinical trial sites in Latin America although the entire process complicates Health in all these countries. Patients are enrolled with many improving health outcomes. These patients of course expect to continue with therapy. Despite all best intentions by health care providers and industry, however, the drug may never be approved and/or available for them or for other patients who may have potential benefit.

Latin American countries need to unite in an initiative to improve Health. Health EURORDIS Rare Diseases in Europe is an example of how to approach the issues faced specifically in rare disease. The European Union's 27 countries of varied political and economic agendas coupled with different societal and cultural norms, inadvertently has staggered progress in unifying health. They advocate a Europe‐wide collaboration for a unified market.^1^ Economic initiative need to partner with educational initiatives and improved diagnostics. Perhaps first starting with governments with similar ideals or even country‐specific like the approach used in Ireland.^5^ Partnership with local patient organizations will help disseminate education and advocate to government health agencies the need to increase the range of drugs available to PH patients. As PH is a global problem, collaborations between developed and developing countries will serve to accelerate research and education that will serve a mutually beneficial purpose of improving the quality of life of patients living with PH.

PH physicians and their patients in Latin America and globally need to utilize the existing rare disease networks infrastructure and expertise to advance health. It is difficult to practice medicine to guideline standards in most countries and even more difficult to tackle the multiple political and economic issues as they relate to health. The authors should be commended for their efforts to illustrate the differences in care in their countries and by nudging us to be more active from a global perspective.

## CONFLICT OF INTERESTS

The authors declare that there are no conflict of interests.

## ETHICS STATEMENT

Not applicable.

## References

[1] EURORDIS RDEaiM . Position Paper: breaking the access deadlock to leave no one behind. https://Eurodis.org (2018). Accessed 6 Sept, 2021.

[2] Universal Rights Group. Human rights and the sustainable development goals. https://www.universal-rights.org/30-years-on-demystifying-and-depoliticising-the-right-to-development/ (2021). Accessed 10 Jul, 2021.

[3] Orozco‐Levi M , Cáneva J , Fernandes C , Restrepo‐Jaramillo R , Zayas N , Conde R , Diez M , Jardim C , Pacheco Gallego MC , Melatini L , Valdéz H , Pulido T . Differences in health policies for drug availability in pulmonary arterial hypertension and chronic thromboembolic pulmonary hypertension across Latin America. Pulm Circ. 2021;12(1):e12012. 10.1002/pul2.12012 PMC9053007 35506085

[4] Valverde AB , Soares JM , Viana KP , Gomes B , Soares C , Souza R . Pulmonary arterial hypertension in Latin America: epidemiological data from local studies. BMC Pulm Med. 2018;18(1):106. 10.1186/s12890-018-0667-8 29940945 PMC6019295

[5] Somanadhan S , Nicholson E , Dorris E , Brinkley A , Kennan A , Treacy E , Atif A , Ennis S , McGrath V , Mitchell D , O'Sullivan G , Power J , Lawlor A , Harkin P , Lynch SA , Watt P , Daly A , Donnelly S , Kroll T . Rare Disease Research Partnership (RAinDRoP): a collaborative approach to identify research priorities for rare diseases in Ireland. HRB Open Res. 2020;3:13. 10.12688/hrbopenres.13017.2. 33299965 PMC7702160

